# Prehospital naloxone administration – what influences choice of dose and route of administration?

**DOI:** 10.1186/s12873-020-00366-3

**Published:** 2020-09-05

**Authors:** Ida Tylleskar, Linn Gjersing, Lars Petter Bjørnsen, Anne-Cathrine Braarud, Fridtjof Heyerdahl, Ola Dale, Arne Kristian Skulberg

**Affiliations:** 1 Department of Circulation and Medical Imaging, Norwegian University of Science and Technology, NTNU, Trondheim, Norway; 2 Clinic of Emergency Medicine and Prehospital Care, St. Olavs hospital, Trondheim University Hospital, Trondheim, Norway; 3 Department of Alcohol, Tobacco and Drugs, Norwegian Institute of Public Health, Oslo, Norway; 4 Division of Prehospital Services, Oslo University Hospital, Oslo, Norway; 5 The Norwegian Air Ambulance Foundation, Oslo, Norway; 6 Department of Research and Development, St. Olavs hospital, Trondheim University Hospital, Trondheim, Norway

**Keywords:** Emergency medical services, Naloxone, Heroin, Mortality, Drug Administration Routes, Drug Overdose/drug therapy, Administration and dosage

## Abstract

**Background:**

Amidst the ongoing opioid crisis there are debates regarding the optimal route of administration and dosages of naloxone. This applies both for lay people administration and emergency medical services, and in the development of new naloxone products.

We examined the characteristics of naloxone administration, including predictors of dosages and multiple doses during patient treatment by emergency medical service staff in order to enlighten this debate.

**Methods:**

This was a prospective observational study of patients administered naloxone by the Oslo City Center emergency medical service, Norway (2014–2018). Cases were linked to The National Cause of Death Registry. We investigated the route of administration and dosage of naloxone, clinical and demographic variables relating to initial naloxone dose and use of multiple naloxone doses and one-week mortality.

**Results:**

Overall, 2215 cases were included, and the majority (91.9%) were administered intramuscular naloxone. Initial doses were 0.4 or 0.8 mg, and 15% of patients received multiple dosages. Unconscious patients or those in respiratory arrest were more likely to be treated with 0.8 mg naloxone and to receive multiple doses. The one-week mortality from drug-related deaths was 4.1 per 1000 episodes, with no deaths due to rebound opioid toxicity.

**Conclusions:**

Intramuscular naloxone doses of 0.4 and 0.8 mg were effective and safe in the treatment of opioid overdose in the prehospital setting. Emergency medical staff appear to titrate naloxone based on clinical presentation.

## Background

There has been an ongoing rise in deaths from opioids [1] and in 2017, the U.S. Department of Health and Human services declared this a public health emergency [2]. In response to the opioid overdose epidemic, take-home naloxone programs and new naloxone formulations for opioid overdose reversal have been developed [1, 3, 4]. There is no agreement on the optimal route of administration or dosages, leaving no established best practices when naloxone is administered in the community. After discussions within an advisory committee to the U.S. Food and Drug Administration (FDA), the committee narrowly voted to increase the minimum recommended naloxone exposure of 0.4 mg for novel products entering the market, without specifying an acceptable dose [5]. Currently, the FDA is still considering this advice and no formal recommendation has been made regarding minimal naloxone dose [6].

### Importance

When investigating new formulations such as nasal naloxone, one needs to know what doses and routes new formulations should be compared to. It is not only take-home naloxone programs that lack uniform guidelines and best practices. Naloxone has been available to emergency medical services (EMS) since the 1970s, the recommended initial dosage range is wide, ranging from 0.4 to 2 mg naloxone hydrochloride [1, 7], and the optimal route and dosages have not been scientifically established. Traditionally, naloxone has been administered both intravenously (IV), intramuscularly (IM) and subcutaneously, with different policies regarding dosages and routes of administration across services and countries [1]. In the treatment of respiratory arrest, rapid restoration of the patient’s own breathing is vital, but the price to pay for aggressive naloxone treatment is eliciting opioid withdrawal symptoms [8]. This should not be ignored as a minor issue. Withdrawal symptoms can lead to further drug seeking and may make patients reject further necessary follow-up [9]. Consequently, there is a need for more evidence on the most effective route of administration and dosages that do not induce opioid withdrawal symptoms but that also ensure no rebound opioid toxicity.

### Goal of this investigation

We examined characteristics of naloxone administration among patients attended by the largest EMS in Norway between 2014 and 2018, including a) route of administration, b) dosage and c) number of doses administered at each EMS attendance. We estimated the putative associations between naloxone dose and sex, age, place of attendance and vital signs. We estimated the likelihood of administration of multiple naloxone dosages in a single EMS attendance as a function of initial dose, sex, age, place of attendance and vital signs. We examined transport rates following EMS treatment and the one-week mortality rate to provide safety data for clinical practice.

## Methods

### Study design

This was a 5-year observational study of patients treated with naloxone by the Oslo City Center EMS. Participants were prospectively included between June 1st, 2014 and December 31st, 2018 and were thereafter followed through the National Cause of Death Registry until December 31st, 2018. Data from January 1st, 2014 to May 31st, 2014, were collected retrospectively and registered anonymously with no matching against the death registry.

### Setting

During the study, Norway had a population of 5.3 million people [10] and a high rate of fatal opioid overdoses [11]. The most commonly used illicit opioid was heroin. Although a range of other opioids are misused, fentanyl played a minor role in the current drug market [11]. The capital Oslo had 690,000 inhabitants [12] and the EMS covering the city center of Oslo (Oslo City Center EMS) was the largest service and attended the majority (67%) of the city’s overdose cases. During the study, Oslo City Center EMS were set up with ground ambulances only. Non-transporting response vehicles, physician manned rapid response unit, and other more specialized units were organized outside of the city center EMS but could assist the city center units as needed. The ground ambulances were staffed by personnel with competence ranging from emergency medical technicians with two years of high school education followed by two years of clinical practice, to paramedics with a three-year bachelor’s degree. The recommended local management of suspected opioid overdose was assisted ventilation and naloxone administration. The suggested therapy was the administration of 0.4 mg to 0.8 mg IM naloxone followed by 0.4 mg IV. Dosing should be based on clinical presentation, and a suggestion was made to consider 0.8 mg IM for patients weighing more than 70 kg. Further titration with 0.4 mg IV up to a total dose of 2 mg was recommended if respiration and consciousness were not restored [13]. The EMS administered naloxone hydrochloride in formulations for injection of 0.4 mg/mL. Oslo had a public safe injection facility located in the center where drug users could inject illicit drugs provided by themselves. The facility provided the drug users with sterile equipment, information about safe drug injecting techniques, and presence of health care professionals. The facility staff monitored the users and called for professional assistance when needed. Staff were typically nurses or social workers trained in basic airway management.

### Participants

Patients were included if they were 18 years or older and naloxone was administered by the Oslo City Center EMS. According to European resuscitation guidelines, treatment with naloxone were not recommended for patients with opioid-induced cardiac arrest, and this was also the guideline in the Oslo City Center EMS [7]. However, one patient received naloxone prior to the recognition of cardiac arrest and since this was an observational study with the aim of documenting all EMS use of naloxone, the individual case was included in our material. The Committee for Research Ethics approved a procedure with waived consent with the opportunity to actively withdraw from the study. Patients received oral and written information about the study at the time of treatment by EMS. All patients were included except those who asked to be withdrawn from the study on site or contacted the study team later by phone.

### Data sources

The Oslo EMS used paper-based medical records. Records for included patients were copied and filed in a separate system. A trained research nurse manually entered data from the records into a database. Patient sex, patient age, place of attendance, and naloxone doses and their routes of administration were registered. Clinical variables such as respiration rate (RR) and consciousness reported as a Glasgow Coma Scale (GCS) score, both before and after EMS treatment with naloxone, were also recorded. Prior to analysis, the key data were verified by two researchers against the original records. Missing data were not imputed. The data management system used was VieDoc version 4 (PCG Solutions, Uppsala, Sweden). For each patient, the first event after June 1st, 2014, was defined as the index episode, and all subsequent episodes were classified as “repeated episodes”. National identity numbers were used to link episodes. The date and cause of death were retrieved from the National Cause of Death Registry.

### Statistics

Statistical analyses were conducted in STATA 15.1. Descriptive statistics were used to describe the route of naloxone administration, naloxone dosages and the number of doses administered during EMS attendance. We used univariate and multivariable logistic regression analyses to examine 1) the associations between naloxone dose and patient sex, patient age, place of attendance and vital signs and 2) the associations between multiple naloxone doses (≥2) during an EMS attendance and patient sex, patient age, place of attendance, vital signs and initial naloxone dose. The regression analyses only included cases with a valid national identity number, as this allowed for accounting of repeated events by including identity as a cluster variable in the models. Odds ratios (ORs) and adjusted ORs (AORs) with 95% confidence intervals (CIs) were reported.

We reported transport rates following EMS treatment. These rates included being left at the scene or transported to a hospital, a primary care accident and emergency outpatient clinic, or other places such as home or addiction treatment facilities. We examined one-week mortality after treatment. The date of death was retrieved from the National Cause of Death Registry. The time of death was not available. Deaths registered on the same date as EMS treatment were defined as “day 0” and deaths the following date as “day 1”. To estimate one-week mortality, we used deaths that occurred on day 0 through day 7.

### Measures

The dependent variable in the first logistic regression (Model 1) was IM naloxone at doses of 0.4 and 0.8 mg, and 0.4 mg naloxone was the reference category. Only 3.6% received naloxone by other dosages and 7.4% via other routes; therefore, we excluded these from the analysis. In Model 1, the following explanatory variables were included: patient sex, patient age, GCS and respiration rate at presentation to the EMS and if the overdose was attended at the safe injection facility. Low GCS score and respiration rate are part of the classic opioid overdose triad and have been shown to influence the choice of naloxone dose [14, 15]. Other patient characteristics, such as sex, have also been found to influence the choice of naloxone dose in one study [15]. Age and treatment at the safe injection facility were included as part of an exploratory analysis.

The dependent variable in the second logistic regression model (Model 2) was multiple doses of naloxone (≥2 doses). The reference category was a single dose only. In Model 2, the following explanatory variables were included: initial naloxone dose, patient sex, patient age, GCS and respiration rate at first evaluation and if the overdose occurred at the safe injection facility. To ensure that missing data were not deleted listwise in the logistic regression analysis, a category for missing responses for variables with incomplete recordings (no valid reports) was included.

## Results

**Fig. 1:**
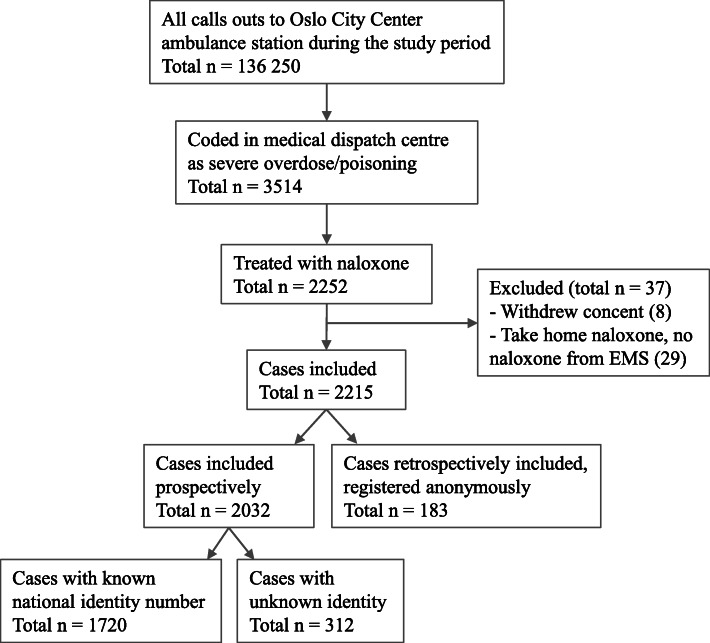
Flowchart of inclusion and exclusion in the study

Between 2014 and 2018, 2215 cases were treated with naloxone by the Oslo City Center EMS (Fig. 1). Eight patients declined participation. Twenty-nine patients were excluded because they were administered naloxone by others prior to EMS attendance, and no further naloxone administration was needed.

The mean age of the patients was 38.3 years, and 77.1% were men (Table 1). The median GCS was 4, and the median respiratory rate was 7 breaths/minute. As shown in Table 1, the safe injection facility was the place of attendance in 33.5% of the patients. The remaining cases (not shown) were attended in public places (50.1%), private homes (7.3%), shelters/other facilities for people using drugs (6.5%), and other places such as hotels and public transport (2.6%).

**Table 1:** Cases in which naloxone was administered by Oslo City Center emergency medical services between 1st of January 2014 and 31st of December 2018

	Total100% (***n*** = 2215)	No valid report % (n)
Known national identity number (% (n))	77.6 (1720)	22.3 (495)
Men (% (n))	77.1 (1707)	0.7 (15)
Age (mean (SD))	38.3 (11.2)	13.5 (298)
Glasgow Coma Scale (median (min-max))	4/15 (3–15)	8.5 (188)
Respiration rate/minute (median (min-max))	7 (0–40)	12.5 (276)
Attended in safe injection facility (% (n))	33.5 (743)	0 (0)

Intramuscular injection was the most common route of naloxone administration (Table 2), as 91.9% (*n* = 2035) of the 2215 cases received this as their initial treatment. Only a minority of patients were treated with IV naloxone; 1.9% (*n* = 41) were treated with IV alone, and 3.8% (*n* = 84) were administered IV naloxone after the administration of an IM dose. A minority of patients (2.5%) were administered naloxone by other routes, such as intranasal or subcutaneous routes. The use of IV naloxone as the initial treatment became less frequent during the study period, decreasing from 50 cases in 2014 to only two cases in 2018.

**Table 2:** Routes of administration and dose of naloxone in 2215 suspected cases of opioid overdose and subsequent administration of naloxone after the initial dose

Initial naloxone treatment	% (n)	Subsequent naloxone administration, % (n)
**Total**	100 (2215)	15.0 (332)
**IM only**	91.9 (2035)	15.6 (318)
0.4 mg	39.9 (811)	16.5 (134)
0.8 mg	56.5 (1150)	15.0 (172)
Other doses < 0.8 mg	3.5 (72)	
Other doses > 0.8 mg	0.1 (2)	
**IV only**	1.9 (41)	9.8 (4)
0.4 mg	75.6 (31)	
0.8 mg	17.1 (7)	
Other doses < 0.8 mg	7.3 (3)	
**IM and IV**	3.8 (84)	2.4 (2)
0.4 IM + 0.4 IV	17.9 (15)	
0.8 IM + 0.4 IV	65.5 (55)	
0.8 IM + 0.8 IV	10.7 (9)	
Other doses > 0.8 mg	6.0 (5)	
**Other**	2.5 (55)	14.6 (8)

IM = intramuscular, IV = intravenous

Among those treated with IM naloxone (*n* = 2035), the most common dose was 0.8 mg (56.5%), followed by 0.4 mg (39.9%). Only 3.6% (*n* = 74) received IM naloxone in other doses.

Overall, only 15.0% (*n* = 332) of the 2215 cases were administered a second or third dose of naloxone. The majority (82.0%) of these 332 cases were treated with only one additional dose. Among those administered multiple doses (≥2), 51.5% received IV and 48.5% received IM naloxone.

Among the 2215 cases, the total administered naloxone dose was 0.4 mg for 33.0% of the patients, 0.8 mg for 51.2% of the patients and more than 0.8 mg for 12.7% of the patients. 3.1% received other doses less than 0.8 mg. This included the initial and subsequent doses of titration. Only 1.0% of patients received ≥2 mg naloxone in total, and the maximum dose used was 3.0 mg. The mean total dose of naloxone in patients with respiratory arrest or cyanosis was 0.8 mg.

### Naloxone dose and its associations with clinical variables

Of the 2215 cases, 1720 cases had a valid national identity number (Table 1). This subgroup comprised 869 individuals; 76.3% were men, and the mean age was 38.6 years. The majority of these individuals (66.0%, *n* = 574) were only attended once. Two attendances were registered in 15.4% of these patients (*n* = 134), while 18.5% of these patients (*n* = 161) were attended three times or more, with a maximum of 27 attendances in the same individual.

The majority (89.0%, *n* = 1530) of the 1720 cases with a valid national identity number were treated with either 0.4 mg or 0.8 mg IM naloxone. Among these patients (Model 1, Table 3), unconscious patients with GCS scores of 3 or 4 to 9 were seven- and four-times more likely to be administered 0.8 mg naloxone than those who were awake (GCS 15). Compared to patients with a respiratory rate of ≥9 breaths/minute, those with respiratory arrest or a respiratory rate of 1–8 breaths per minute were three- and two-times as likely to be treated with 0.8 mg naloxone, respectively. Furthermore, men were more than twice as likely as women to be administered a dose of 0.8 mg. Those attended at the safe injection facility were 40% less likely to receive 0.8 mg naloxone than patients treated at other locations.

**Table 3:** The putative associations between intramuscular naloxone dose (0.4 mg vs. 0.8 mg) and sex, age, vital signs and place of attendance (*n* = 1530), Model 1

	0.4 mg 100% (***n*** = 657)	0.8 mg 100% (***n*** = 873)	Unadjusted OR (95% CI)	Adjusted OR (95% CI)
**Sex**				
Women	30.8 (202)	18.6 (162)	ref	ref
Men	69.3 (455)	81.4 (711)	2.0^***^ [1.5, 2.5]	2.2^***^ [1.7, 2.9]
**Age (years)**				
< 30	24.2 (159)	23.1 (202)	ref	ref
30–49	58.5 (384)	59.7 (521)	1.1 [0.8, 1.4]	1.2 [0.9, 1.5]
≥ 50	17.4 (114)	17.2 (150)	1.0 [0.7, 1.5]	1.3 [0.9, 1.8]
**Glasgow Coma Scale**				
3/15	27.7 (182)	56.5 (493)	9.1^***^ [5.2, 16.2]	7.1^***^ [3.8, 13.1]
4–9/15	21.6 (142)	19.8 (173)	4.1^***^ [2.2, 7.5]	4.0^***^ [2.1, 7.5]
10–14/15	33.8 (222)	13.8 (120)	1.8^*^ [1.0, 3.2]	1.8 [1.0, 3.2]
15/15	8.2 (54)	1.8 (16)	ref	ref
No valid report	8.7 (57)	8.1 (71)	4.2^***^ [2.2, 8.0]	3.8^***^ [2.0, 7.4]
**Respiration rate**				
0/min	7.2 (47)	20.6 (180)	5.1^***^ [3.5, 7.6]	3.4^***^ [2.2, 5.3]
1–8/min	35.5 (233)	43.0 (375)	2.2^***^ [1.7, 2.7]	1.7^***^ [1.3, 2.2]
≥ 9/min	44.9 (295)	25.2 (220)	ref	ref
No valid report	12.5 (82)	11.2 (98)	1.6^**^ [1.1, 2.3]	1.6^*^ [1.1, 2.3]
**Place of attendance**				
Safe injection facility	41.1 (270)	37.5 (327)	0.9 [0.7, 1.1]	0.6^***^ [0.5, 0.8]
All other locations	58.9 (387)	62.5 (546)	ref	ref

Logistic regression analysis was used. Identity was included as a cluster variable to account for individuals that had repeated overdose events and were included multiple times during the study period. OR = odds ratio, 95 CI = 95 confidence interval. ^*^ *p* < 0.05, ^**^ *p* < 0.01, ^***^ *p* < 0.001

### Multiple naloxone dosages and their associations with clinical variables

Overall, multiple doses (≥2) of naloxone during one EMS attendance were administered in 14.8% (*n* = 227) of the 1530 patients with a valid national identity number who received either 0.4 mg or 0.8 mg IM naloxone. Among these cases (Model 2, Table 4), unconscious patients with GCS scores of 3 or 4 to 9 were seventeen- and eight-times more likely to be administered multiple doses than those who were awake. Compared to patients with a respiratory rate of ≥9 breaths/minute, patients with respiratory arrest were twice as likely to be treated with multiple doses. Furthermore, men were almost twice as likely as women to receive multiple dosages. Those attended at the safe injection facility were 80% less likely to be treated with multiple dosages than patients treated at other locations. Finally, those treated with an initial naloxone dose of 0.8 mg were 60% less likely to receive multiple doses than patients treated with an initial dose of 0.4 mg naloxone.

**Table 4:** The likelihood of multiple-dose administration of naloxone during a single EMS attendance as a function of sex, age, vital signs, place of attendance and dose (n = 1530), Model 2

	Single dose 100% (***n*** = 1303)	Multiple doses 100% (n = 227)	Unadjusted OR (95% CI)	Adjusted OR (95% CI)
**Sex**				
Women	24.7 (322)	18.5 (42)	ref	ref
Men	75.3 (981)	81.5 (185)	1.5 [1.0, 2.2]	1.8^**^ [1.2, 2.6]
**Age (years)**				
< 30	23.0 (300)	26.9 (61)	ref	ref
30–49	59.5 (775)	57.3 (130)	0.8 [0.6, 1.2]	1.0 [0.7, 1.5]
≥ 50	17.5 (228)	15.9 (36)	0.8 [0.5, 1.3]	1.1 [0.6, 1.9]
**Glasgow Coma Scale**				
3/15	40.5 (528)	64.8 (147)	9.5^***^ [2.3, 39.2]	17.1^***^ [3.9, 75.0]
4–9/15	21.2 (276)	17.2 (39)	4.8^*^ [1.1, 20.5]	7.8^**^ [1.8, 34.4]
10–14/15	24.6 (321)	9.3 (21)	2.2 [0.5, 9.7]	2.7 [0.6, 11.9]
15/15	5.2 (68)	0.9 (2)	ref	ref
No valid report	8.4 (110)	7.9 (18)	5.6^*^ [1.2, 24.9]	7.9^**^ [1.7, 36.9]
**Respiration rate**				
0/min	13.8 (180)	20.7 (47)	1.6^*^ [1.1, 2.5]	1.9^*^ [1.2, 3.2]
1–8/min	39.6 (516)	40.5 (92)	1.1 [0.8, 1.6]	1.0 [0.7, 1.5]
≥ 9/min	34.1 (444)	31.3 (71)	ref	ref
No valid report	12.5 (163)	7.5 (17)	0.7 [0.4, 1.1]	0.8 [0.4, 1.4]
**Place of attendance**				
Safe injection facility	43.1 (562)	15.4 (35)	0.2^***^ [0.2, 0.4]	0.2^***^ [0.1, 0.3]
All other locations	56.9 (741)	84.6 (192)	ref	ref
**Initial naloxone dose**				
0.4 mg IM	42.1 (549)	47.6 (108)	ref	ref
0.8 mg IM	57.9 (754)	52.4 (119)	0.8 [0.6, 1.1]	0.4^***^ [0.3, 0.5]

Logistic regression analysis was used. Identity was included as a cluster variable to account for individuals that had repeated overdose events and were included multiple times during the study period. IM = intramuscular naloxone. EMS = emergency medical service, OR = odds ratio, 95 CI =95 confidence interval. ^*^ p < 0.05, ^**^ p < 0.01, ^***^ p < 0.001

### Transport rates

The majority (57.1%) of the 2215 patients were left at the scene (Table 5), 28.1% were taken to the Oslo Accident and Emergency Outpatient Clinic, 12.9% were hospitalized and 1.9% were transported to other places. There was one death during EMS treatment of all the cases in our material. The patient was administered naloxone during advanced cardiac life support and died despite resuscitation efforts, hence not represented in either of the transport categories above. In the subsample of patients left on the scene (*n* = 1264), 50.4% were left without medical supervision, while 49.6% were left at the safe injection facility or other health services such as nursing homes. For patients left on the scene, the average time for EMS attendance was 32.7 min. Whether the patient was transported from the scene following treatment was not significantly associated with the initial dose either in the univariate logistic regression analysis (OR 1.1, 95% CI 0.9–1.3), or after adjusting for individual characteristics and vital signs (AOR 1.1, 95% CI 0.9–1.5). However, patients transported following treatment were 70% more likely to have been treated with multiple doses of naloxone both in unadjusted analysis and after adjusting for individual characteristics and vital signs (AOR 1.7, 95% CI 1.2–2.3).

**Table 5:** Transport rates after naloxone treatment

Information on transport	100% (n = 2215)
**Left at the scene**	57.1 (1264)
Safe injection facility or health service	49.6 (627)
Public place, homes, shelters and other places	50.4 (637)
**Accident and Emergency Outpatient Clinic**	28.1 (623)
**Hospitalized**	12.9 (286)
**Transported home, to addiction treatment facilities or other places**	1.9 (41)
**Died**	0.05 (1)

### One-week mortality

Among the 1720 episodes between June 1st, 2014 and December 31st, 2018 with a valid national identity number, there were 10 deaths within the first week after EMS treatment. The crude one-week mortality rate was 5.8 per 1000 episodes. Seven deaths were drug-related deaths, while three patients died from natural causes.

Among the seven cases of drug related deaths, six deaths were classified as unintended poisoning and one as suicide by intentional poisoning with heroin; five men and two women with median age of 45 years (min 37, max 60). All patients underwent autopsy and had heroin confirmed as their main opioid at time of death. None of the patients died on day they were provided with clinical assistance from EMS (day 0), three died on day 1, two died on day 2, and two died within the next five days (days 3 to 7). All had been left at the scene after the last known naloxone treatment prior to their death. The overall one-week mortality rate for drug-related deaths was 4.1 per 1000 episodes and 6.9 per 1000 episodes for patients left at the scene by the EMS.

Three patients died from natural causes: a 96-year-old nursing home patient, a 76-year-old patient in palliative care, and a 62-year-old complex medical patient in home care that died from chronic obstructive pulmonary disease and acute lower respiratory infection after five days of hospitalization.

## Discussion

The majority of included patients were administered IM naloxone injections of 0.4 or 0.8 mg. Multiple doses (≥2) were administered in 15% of cases. Patients who were unconscious or in respiratory arrest were more likely to be treated with 0.8 mg naloxone than 0.4 mg naloxone and more likely to receive multiple doses. Patients who were attended at the safe injection facility were less likely to be treated with 0.8 mg naloxone and less likely to be treated with multiple doses. An initial dose of 0.8 mg naloxone reduced the likelihood of multiple doses by 60%. Patients were left on the scene in more than half of the cases. The one-week mortality rate for drug-related deaths for patients was 4.1 per 1000 episodes. None of the deaths were due to rebound opioid toxicity.

The naloxone dosage observed in the present study was similar to what was found in an Austrian study from 2000 [14]. We observed a dose reduction in Oslo from 1.2 mg 20 years ago to 0.8 mg today in overdoses with respiratory arrest or cyanosis [16]. This reduction is explained by a change in clinical practice, where fewer patients are administered IV naloxone [16]. During our 5-year observation period, only 3.8% of the cases received this treatment. We speculate that the reduced use of IV naloxone is related to the time it takes for establishing IV access [17] and its high rate of adverse events [16]. Anecdotally, staff report reduced opioid withdrawal and increased cooperation of the patients after reversal with antidote through IM injections alone.

In line with a previous study [15], there was an inverse relationship between level of consciousness or respiratory rate and naloxone dosage. Men were twice as likely to be treated with the higher dose than women. This has also been shown previously [15] and might be related to guidelines emphasizing dosing depending on body size [13]. The need for multiple dosages was associated with the same factors as those associated with the initial dose, and an initial dose of 0.8 mg reduced the probability of the administration of multiple dosages by 60%. This indicates that EMS staff use their clinical judgment to titrate naloxone dosing according to clinical presentation and treatment response.

Interestingly, patients at the safe injection facility were often treated with the lower naloxone dosage (0.4 mg) and were less likely to receive a second dose than patients at other locations, despite presenting in deep coma and respiratory arrest. The staff at the facility does not administer naloxone but manages patients with bag-mask ventilation. The lower dose may be a consequence of patients being ventilated while waiting for the EMS and therefore becoming less hypoxic. The facility is also a well-organized work environment and allows the EMS to start lower in their titration of dosages and give this dose time to work. Patients treated in the safe injection facility were also more likely to be left at the premises, probably due to the facility offering post-overdose monitoring and counseling [18].

A large proportion of the patients were left on site. All patients were offered transport to further care after naloxone treatment by the EMS, but many declined this offer. This is a recognized challenge worldwide [16, 19]. The criteria for being left on site included having an appropriate level of consciousness and the capacity to give informed consent. No patients were transported against their will. Three patients died of overdose on day 1 after they had received EMS naloxone. These patients were alive longer than the expected duration of action of the naloxone and are therefore unlikely to be rebound opioid intoxications. This shows that the naloxone dosing regimens used, combined with an average observation time of 32 min, are safe in terms of immediate mortality. These findings are in keeping with previous studies on discharging patients on site after naloxone treatment [19–22].

Opioid overdoses are known risk factors for early death [23]. Repeated overdose prevention and addiction treatment should therefore be a priority. In this study, the one-week mortality rate after being left at the scene was 6.9 per 1000 episodes. This is higher than the 0.8 per 1000 episodes previously reported in a review of the risk for rebound opioid toxicity after naloxone treatment for patients left on the scene [19]. On the other hand, in a study of 2241 patients discharged after naloxone treatment, the 48-h mortality was reported to be 5.8 per 1000 episodes when counting all overdose-related deaths, not just those attributed to rebound opioid toxicity [21]. This might indicate a need to widen the perspective beyond solely focusing on rebound toxicity but also on the risk of death by repeated overdoses for patients being left on the scene after treatment with naloxone. Deaths from new overdoses must be considered preventable events, and efforts must be made to provide appropriate interventions. An Australian study of 3921 overdoses reported 11 deaths from new overdoses within one week after EMS treatment. Nine had been brought to the hospital, of which three self-discharged and died within 24 h of EMS attendance [22]. Being brought to a hospital or a healthcare facility is therefore not necessarily protective, but probably depends on what further treatments are offered during hospitalization.

A dose of naloxone of up to 0.8 mg has been found to be sufficient in the community setting where illicitly manufactured fentanyl circulates and for use by EMS when treating fentanyl overdoses [24, 25]. For patients with a higher level of consciousness and higher respiratory frequency, a dose of 0.4 mg could be a safe alternative. These findings are relevant in the discussions around dosages administered through take-home naloxone regimens and for new naloxone formulations.

### Limitations and strengths

Data collection was based on paper records, which limited the number of variables. National identity numbers were not available in 22.3% of the cases. Some of these, 14.1% (*n* = 312) of the total number of cases, consists of individuals who did not disclose their identification to EMS staff. There is a possibility that this group could have different outcomes, including mortality, than those with known national identification number. Furthermore, 8.2% (*n* = 183) of the total number of cases were systematically collected retrospectively without national identification as ethical approval for using national identity numbers was pending in the first five months of the study period. We assume that they do not differ regarding outcomes from the cases with known identity. Data on clinical evaluations after naloxone administration, such as GCS scores and respiratory rate, were missing in a large proportion of the records, which made it difficult to reliably estimate the efficacy of treatment. Local guidelines recommend naloxone dosages based on the patients’ weight which was not recorded in the medical records and could not be included in analyses. Cases of opioid overdose presenting to EMS as cardiac arrest were excluded from this study as naloxone was not routinely administered in these cases. There were few recorded overdoses with fentanyl or other strong synthetic opioids in Norway, and the results are therefore not necessarily generalizable to settings where fentanyl is more frequent. Linking of data with other national registers and better data on follow-up would have improved the study.

A strength of the study was the long observation period of five years. Key demographic variables in the study could be compared with previous reports in Oslo and other countries [19]. The issues with missing data were handled by including missing data as a variable in the models to avoid observations being deleted listwise. Norway has unique national identity numbers, which made it possible to link the data to the National Cause of Death Registry.

## Conclusion

Initial doses of 0.4 to 0.8 mg of IM naloxone appear effective and safe for the treatment of prehospital opioid overdoses. The data support that the emergency medical staff titrates naloxone based on clinical presentation and effect. GCS and respiratory rate stand out as strong predictors for dosing choices by the EMS in Oslo. Even though the risk of rebound opioid toxicity was low, the population in this study had an alarmingly high one-week mortality rate, much higher than previously reported.

## Data Availability

Due to the nature of this research on a vulnerable patient group, participants of this study did not agree for their data to be shared publicly, so supporting data is not available.

## Abbreviations

EMS: Emergency Medical Service
FDA: the U.S. Food and Drug Administration GCS Glasgow Coma Scale
IM: Intramuscular
IV: Intravenous
RR: Respiration rate
OR: Odds ratio
AOR: Adjusted odds ratio
CI: Confidence interval
